# A High-Fat Diet Delays Age-Related Hearing Loss Progression in C57BL/6J Mice

**DOI:** 10.1371/journal.pone.0117547

**Published:** 2015-01-27

**Authors:** Takeshi Fujita, Daisuke Yamashita, Natsumi Uehara, Go Inokuchi, Shingo Hasegawa, Naoki Otsuki, Ken-ichi Nibu

**Affiliations:** Department of Otolaryngology-Head and Neck Surgery, Kobe University Graduate School of Medicine, Kobe, Japan; Oklahoma State University, UNITED STATES

## Abstract

**Objective:**

Age-related hearing loss (AHL), or presbycusis, is the most common sensory disorder among the elderly. We used C57BL/6J mice as an AHL model to determine a possible association between AHL and a high-fat diet (HFD).

**Methods:**

Forty C57BL/6J mice were randomly assigned to a control or HFD group. Each group was divided into the following subgroups: 1-, 3-, 5- and 12-month groups (HFD, n = 5/subgroup; control, n = 5/subgroup). Nine CBA/N-slc mice were also used as a 12-month control (n = 5) or 12-month HFD (n = 4) group. The mice were fed a HFD or normal (control) diet throughout this study. Hearing function was evaluated at 1, 3, 5 and 12 months using auditory evoked brainstem responses (ABRs). Spiral ganglion cells (SGCs) were also counted.

**Results:**

The elevation of ABR thresholds (at 4 and 32 kHz) at 3 and 5 months was significantly suppressed in the HFD group compared with the control groups for C57BL/6J mice. After 12 months, the elevation of ABR thresholds was significantly suppressed in the HFD group at all frequencies for C57BL/6J mice. In contrast, CBA/N-slc mice displayed opposite outcomes, as ABR thresholds at all frequencies at 12 months were significantly elevated in the HFD group compared with the control group. For the C57BL/6J mice at 12 months, SGC numbers significantly decreased in all parts of the cochleae in the control group compared with the HFD groups. In contrast, for the CBA/N-slc mice, SGC numbers significantly decreased, particularly in the upper parts of the cochleae in the HFD group compared with the control groups.

**Conclusions:**

The elevation in ABR thresholds and SGC loss associated with aging in the HFD-fed C57BL/6J mice were significantly suppressed compared with those in the normal diet-fed mice. These results suggest that HFD delays AHL progression in the C57B/6J mice.

## Introduction

Age-related hearing loss (AHL), or presbycusis, affects tens of millions of people worldwide and is characterized by reduced hearing sensitivity and understanding speech, slowed central processing of acoustic information, and impaired localization of sound sources [1]. Risk factors for AHL generally fall into one of the four categories: cochlear aging (individual’s age), environmental (occupational and leisure noise exposure, ototoxic medications, and socioeconomic status), genetic predisposition (sex, race, and specific genetic loci/genes), and health comorbidities (hypertension, diabetes, stroke, and cigarette smoking) [2]. Dyslipidemia have also been reported to be associated with AHL [3][4][5]. However, not all studies succeeded in showing an association between dyslipidemia and sensorineural hearing loss [6].

Several studies using animal models assessed the adverse effects of a high-fat diet (HFD) on AHL in CBA/CaJ mice [7], CD/1 mice [8], and Sprague–Dawley rats [9].While, other study showed that guinea pigs fed HFD had no significant changes in hearing [10].

C57BL/6 mice are known to develop sensorineural hearing loss at a much earlier age than other mice and are useful for studying AHL features [11][12][13]. These mice display a loss of hair cells and spiral ganglion neurons and elevated hearing thresholds by 12 months of age [14]. To date, associations between AHL and HFD feeding are still inconsistent. Therefore, using C57BL/6J mice, we conducted this study to elucidate the associations between HFD and AHL.

## Materials and Methods

### Mice and HFD

Male C57BL/6J mice (age, 8 weeks) and male CBA/N-slc mice (age, 8 weeks) were used in this study. In contrast to C57BL/6 mice, CBA mice maintain good hearing throughout life [15]. Thus, CBA/N-slc mice were used as controls. All animal procedures were approved by the Institutional Animal Care and Use Committee guidelines of Kobe University Graduate School of Medicine (Permit Number: P100401). The animals were maintained under standard animal house conditions. Mice in HFD groups were fed HFD (High Fat Diet 32, CLEA Japan Inc., Tokyo, Japan). Mice in control groups were fed normal diet (CE-2, CLEA Japan Inc., Tokyo, Japan). The data of ingredients in each food are obtained from CLEA Japan Inc. Body weights and venous blood glucose levels, obtained from the tail and measured by Glutest-Ace (Sanwa Kagaku Kenkyusho, Nagoya, Japan), were measured at baseline (pre-treatment), and 1, 3, 5, and 12 months after starting the experiment.

### Experimental protocol

Forty C57BL/6J mice were randomly assigned to a control or HFD group (20 mice/group), and each group was then divided into the following subgroups (5 mice/subgroup for HFD or control groups): 1-, 3-, 5- and 12-month group. Nine CBA/N-slc mice were also assigned to a 12-month control (n = 5) or a 12-month HFD (n = 4) group.

Mouse hearing function in the 1-, 3- and 5-month subgroups was determined by auditory evoked brainstem responses (ABRs) at baseline (pre-treatment) and 1,3 or 5 months after starting the experiment. Ears for which ABR thresholds exceeded 50 dB SPL at baseline were excluded from this experiment. For the C57BL/6J mice in the 12-month subgroup and the CBA/N-slc mice in the 12-month subgroup, hearing function was assessed by ABR at 12 months after starting the experiment. After the last ABR measurements, the mice were immediately euthanized by cervical dislocation and cochleae were removed.

### Auditory brainstem responses

ABR measurements were made for both ears of each mouse. Mice in the 12-month subgroups had ABR measurements for the right ear only. Prior to these measurements, the mice were anesthetized by intraperitoneal injection of midazolam (10 mg/kg), medetomidine (37.5 μg/kg), and butorphanol tartrate (0.5 mg/kg). ABR measurements were measured using waveform storing and stimulus control with Scope software incorporated in a PowerLab system (PowerLab2/26; AD Instruments, Castle Hill, Australia).

Sound stimuli were produced using a coupler type speaker (ES1spc; Bio Research Center, Nagoya, Japan) inserted into the external auditory canal of a mouse. Tone burst stimuli, with a 0.2 ms rise/fall time (cosine gate) and 1 ms flat segment at frequencies of 4, 8,16, and 32 kHz, were generated by RPvds (Tucker-Davis Technologies, FL), and the sound pressure level was specified by a sound generator and attenuation Real-Time Processor and Programmable Attenuator (RP2.1and PA5; Tucker-Davis Technologies, FL). Sound level calibrations were made using 1/4” microphone, preamp and main microphoneamp (NA-42; Rion Co., LTD., Tokyo, Japan).

For recording, stainless steel needle electrodes were placed at the vertex and ventrolateral to the left and right ears. In general, ABR waveforms were recorded for 12.8 ms at a sampling rate of 40,000 Hz using 50–5000 Hz bandpass filter settings. Two hundreds fifty-six stimuli, delivered at 9 Hz, were averaged to obtain a waveform. ABR waveforms were recorded in 5-dB SPL intervals down from a maximum amplitude until no waveform could be visualized. Threshold was defined as the lowest intensity of stimulation that yielded a waveform, repeatable at least two times based on an identifiable ABR wave I or IV, whichever demonstrated greater sensitivity.

### Histological preparations

After the last ABR measurements were performed under deep anesthesia, the temporal bones were immediately removed and transferred to 4% paraformaldehyde in 0.1 M phosphate-buffered saline (pH 7.4). Under a dissecting microscope, the round and oval windows and the cochlear capsule near the apex were opened, followed by gentle local perfusion of 4% paraformaldehyde from the apex. The tissues were kept in fixative at 4°C for 24 h. After overnight fixation, cochleae were decalcified with 10% ethylenediaminetetraacetic acid disodium salt dihydrate (pH 7.0, Muto Pure Chemicals Co. Ltd., Tokyo, Japan) at room temperature for 2 days. Cochleae were dehydrated through a graded ethanol series and xylene, embedded in paraffin, and then sectioned at 3.0 μm thickness in the midmodiolar plane.

### Morphological analysis

Cochlea sections on slides were stained with hematoxylin (Muto Pure Chemicals Co. Ltd.) and eosin (Wako Pure Chemicals Industries Ltd., Osaka, Japan) (HE staining) to study their structures. Cochlear specimens were examined using a light microscope system (BZ-8100, Keyence, Osaka, Japan) and saved as digital images.

### Spiral ganglion cell counts

For this study, a cochlea was divided into three half turns (basal, upper basal, and apical). Morphometric assessments of SGCs were made for each cochlear turn on hematoxylin and eosin-stained sections. Cochlear specimens were observed and photographed using a BZ-8100 light microscope and digital images were saved. The areas of Rosenthal’s canal and the cochlear turns were quantified by measuring their cut surfaces using a BZ-H1AE, microscope analysis software (Keyence, Osaka, Japan). All neurons that met the size and shape criteria to be considered type I SGCs within each profile of Rosenthal’s canal were counted for each cochlear turn. SGC density was determined as the number of cell nuclei per 10000 μm2 of Rosenthal’s canal. We calculated SGC density as described previously [16] in three mid-modiolar sections at 30 μm apart from each cochlea, with the average value used for each mouse.

### Statistical analysis

Results are given as means ± standard errors. The overall effects on ABR threshold shifts, ABR thresholds, and SGC densities were assessed by non-paired t-tests (Stata 11.1, Stata Corp., College Station, TX). P < 0.05 was considered significant.

## Results

### Body weights and blood glucose levels of control and HFD mice

**Fig 1:**
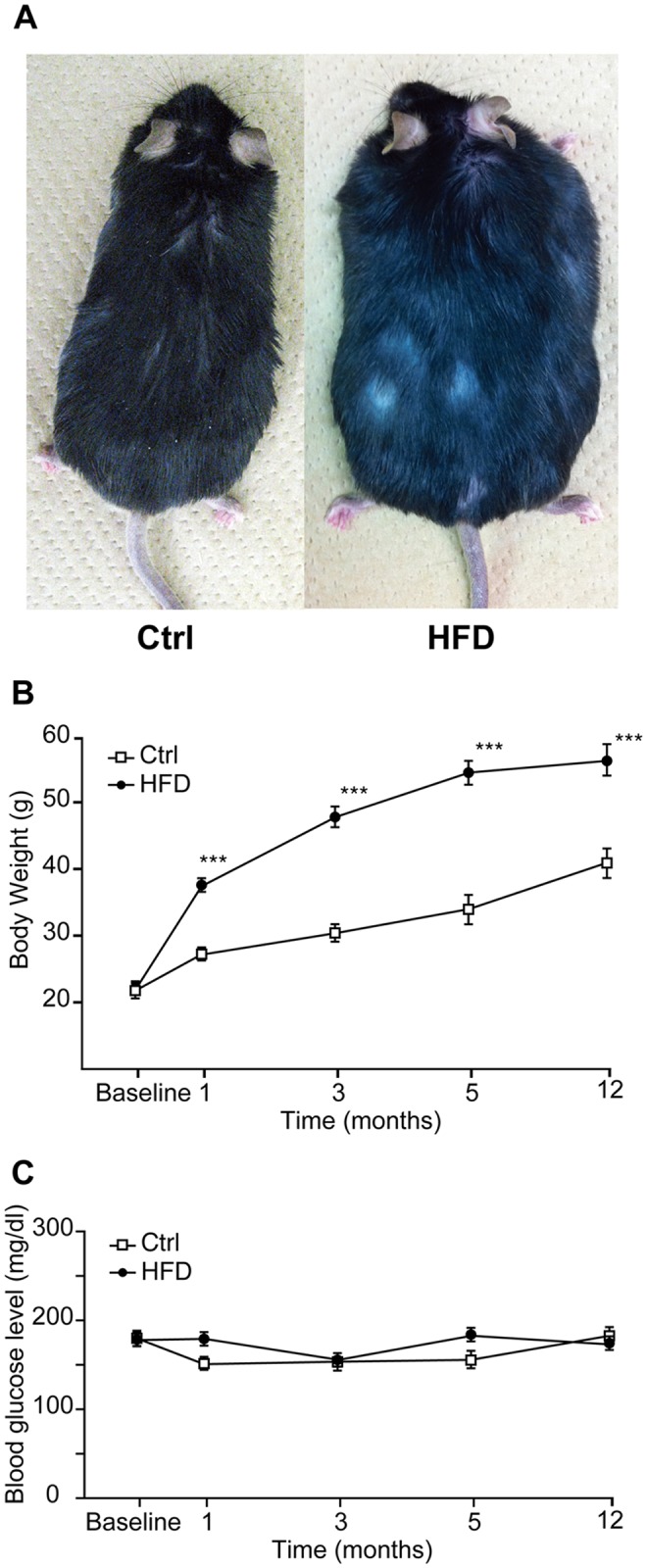
Body weights and blood glucose levels of C57BL/6J mice. (A) Appearances of control diet- and high-fat diet-fed C57BL/6J mice in the 12-month subgroups. Time courses of changes in body weight (B) and blood glucose levels (C) from baseline (n = 15 each for high-fat diet and control diet, 8 weeks old) at 1 month (n = 15 each for high-fat diet and control diet), at 3 months (n = 10 each for high-fat diet and control diet), at 5 months (n = 5 each for high-fat diet and control diet), and at 12 months (n = 5 each for high-fat diet and control diet) from starting the experiment. Results are means ± SEM ***P < 0.001 high-fat diet group vs. control diet group. Ctrl, control diet group; HFD, high-fat diet group.

The changes in body weights and blood glucose levels of the C57BL/6J mice during this study period are shown in Fig. 1. Body weight steadily increased in the control mice throughout the 1-year observation period. In contrast, the HFD mice showed large gains in weight (Fig. 1A). The body weights of the HFD mice significantly increased compared with those of the control mice at 1, 3, 5, and 12 months (Fig. 1B). Blood glucose levels in both groups were not significantly different throughout the experimental period (Fig. 1C).

### Time course for ABR thresholds

**Fig 2:**
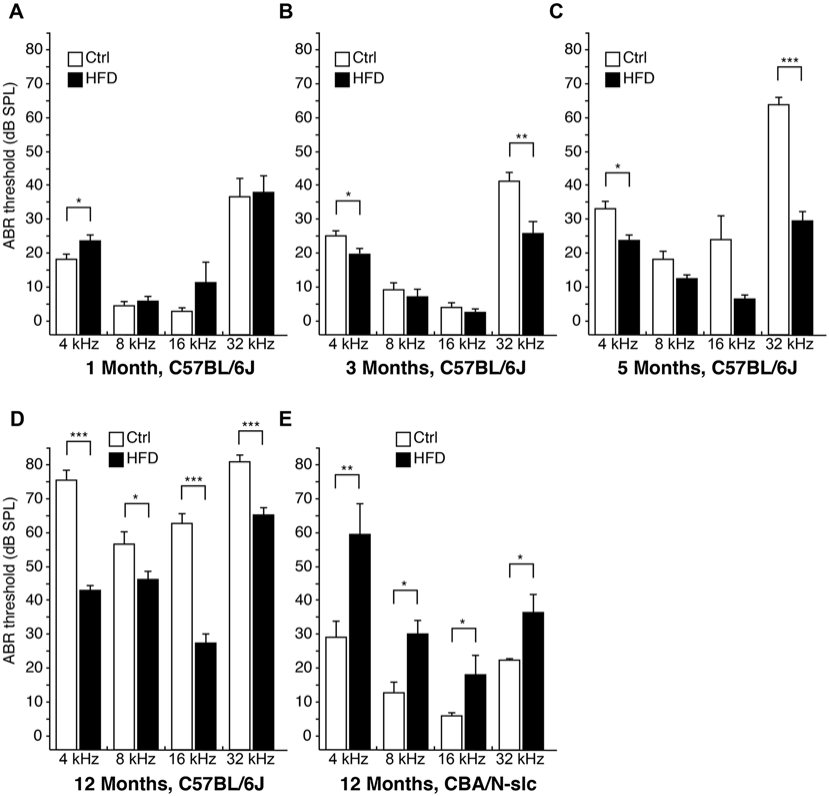
Chronological changes in auditory evoked brainstem response thresholds at 1, 3, 5, and 12 months. Time courses of auditory evoked brainstem response thresholds for the C57BL/6J mice in the high-fat diet and control diet groups at 1 month (A) (n = 5 for each group, 9 ears for the high-fat diet group and 10 ears for the control diet group), at 3 months (B) (n = 5 and 10 ears for each group), at 5 months (C) (n = 5 and 10 ears for each group), and at 12 months (D) (n = 5 and 5 ears for each group). Auditory evoked brainstem response thresholds for the CBA/N-slc (E) mice at 12 months in the high-fat diet group (n = 4 and 4 ears) and the control group (n = 5 and 5 ears). For the C57BL/6J mice, auditory evoked brainstem response thresholds were significantly elevated in the control group compared with the high-fat diet group at 4 and 32 kHz (3 and 5 months) and at all frequencies (12 months). For the CBA/N-slc mice at 12 months, auditory evoked brainstem response thresholds were significantly elevated in the high-fat diet group compared with the control diet group at all frequencies. Results are means ± SEM *P < 0.05; **P < 0.01; ***P < 0.001 high-fat diet group vs. control diet group. Ctrl, control diet group; HFD, high-fat diet group.

At 3 and 5 months of this experiment, ABR thresholds at 4 and 32 kHz were significantly elevated in the control group compared with the HFD group for the C57BL/6J mice (Fig. 2A–C). After 12 months, ABR thresholds at all frequencies were significantly elevated in the control group compared with the HFD group for the C57BL/6J mice (Fig. 2D). In contrast, for the CBA/N-slc mice, ABR thresholds at all frequencies were significantly elevated in the HFD group compared with the control group in the 12-month subgroups (Fig. 2E).

### Histological changes in cochleae

**Fig 3:**
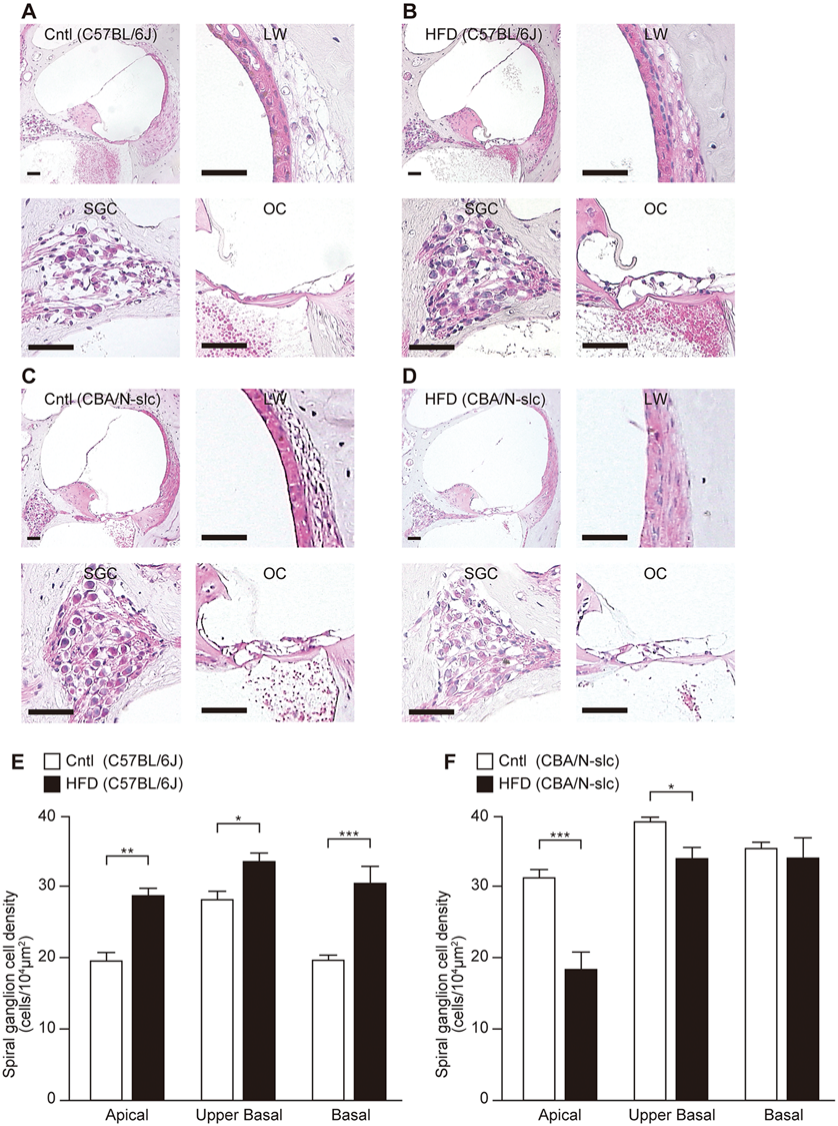
Hematoxylin and eosin staining and densiies of spiral ganglion neurons in 12-month subgroups. Whole cochleae, lateral wall, spiral ganglion cells and organ of Corti in the basal turn of the cochleae (scale bar = 50 μm) of the C57BL/6J and CBA/N-slc mice in the respective 12-month subgroups. Hematoxylin and eosin staining of the cochleae of the C57BL/6J mice (A: control diet, B: high-fat diet) and CBA/N-slc mice (C: control diet, D: high-fat diet). Mean densities of spiral ganglion neurons in the C57BL/6J mice of the 12-month subgroup are shown in (E) (n = 5 each group for high-fat diet and control diet, respectively) and those in the CBA/N-slc 12-month subgroup are shown in (F) (n = 4 for high-fat diet and n = 5 for control diet). Results are means ± SEM *P < 0.05; **P < 0.01; ***P < 0.001 high-fat diet group vs. control diet group. Ctrl, control diet group; HFD, high-fat diet group; LW, lateral wall; SGC, spiral ganglion cells; OC, organ of Cotri.

Preservation of type 1 SGCs was found in all parts of the cochlea in the 12-month HFD subgroup for the C57BL/6J mice. Fig. 3 shows representative sections of Rosenthal’s canal in the basal turn of the cochlea for the control (Fig. 3A) and HFD (Fig. 3B) groups for the C57BL/6J mice and in the apical turn of the cochlea for the control (Fig. 3C) and HFD (Fig. 3D) groups for the CBA/N-slc mice. For the C57BL/6J mice, the average numbers of SGCs in the HFD and control groups after 12 months of being fed the respective diets were 27.6/10000 μm2 and 20.0/10000 μm2 in the apical turn, 33.3/10000 μm2 and 27.1/10000 μm2 in the upper basal turn, and 30.4/10000 μm2 and 19.9/10000 μm2 in the basal turn, respectively (Fig. 3E). For the CBA/N-slc mice, the average SGC numbers in the HFD and control groups after 12 months of being fed the respective diets were 18.0/10000 μm2 and 30.8/10000 μm2 in the apical turn, 33.6/10000 μm2 and 39.0/10000 μm2 in the upper basal turn, and 33.5/10000 μm2 and 35.2/10000 μm2 in the basal turn, respectively (Fig. 3F).

For the C57BL/6J mice, SGC numbers were significantly preserved in all parts of the cochleae in the HFD group compared with the control group. In contrast, for the CBA/N-slc mice, SGC numbers were significantly decreased in the apical and the upper basal turns of the cochleae in the HFD group compared with the control group. By light microscopy, the organs of Corti were well preserved in the HFD group compared with the control group for the C57BL/6J mice (Fig. 3A–B). In contrast, the differences of the organs of Corti were not apparent as the SGC between HFD and control cochleae in CBA/N-slc mice (Fig. 3C–D). No apparent differences were observed in the lateral cochlear wall, including the stria vascularis, between HFD and control cochleae at 1, 3, 5, and 12 months in both mouse strains.

## Discussion

The elevation in ABR thresholds associated with aging and SGC loss in the HFD-fed C57BL/6J mice were significantly suppressed in comparison with those in the normal diet-fed mice. These results suggested that HFD delays AHL progression in the C57B/6J mice. In contrast, the HFD group of the CBA/N-slc mice showed that with aging, ABR thresholds were significantly elevated and SGCs numbers were significantly decreased in comparison with the control group. These findings were consistent with those of previous studies that used the CBA/CaJ mice [7], CD/1 mice [8], and D-galactose-induced aging Sprague–Dawley rats [9]. This indicated that the positive effects of HFD on hearing in the C57BL/6J mice were due to the specific nature of this strain and were not influenced by our rearing environment or the diet followed.

Previous studies reported that HFD induces oxidative stress, mitochondrial damage, and cellular apoptosis in the inner ear of CD/1 mice [8] and Sprague–Dawley rats [9]. Although no known reports have investigated hearing in HFD-fed C57BL/6 mice, several studies have reported significant sensory nerve conduction velocity and motor nerve conduction velocity deficits after feeding HFD to the C57BL/6 mice [17] [18]. In contrast, Guilford et al. reported an increase in motor nerve conduction velocity after HFD feeding to the C57BL/6 mice [19]. The C57BL/6J mouse clearly becomes obese despite an increase in activity levels by HFD. Brownlow et al. reported that HFD-fed obese C57BL/6J mice are nearly three times as active as A/J mice [20]. These mean that the HFD-fed C57BL/6 mouse has specific physiological features different from those of the other strains.

The ingredients in HFD (HFD32) are different from those in the normal diet (CE-2). The main components of the two chows are shown in Table 1. Most components are more abundant in the normal diet than in HFD. Only five components, crude fat, energy, copper, vitamin E, and folic acid, are dominant in HFD in comparison with in the normal diet. Of these components, vitamin E, which has antioxidant and anti-inflammatory properties [21], is three times higher in HFD than in the normal diet. Combination therapy with antioxidants, including vitamin E could reduce noise-induced hearing loss [22] and prevent AHL in C57BL/6 mice [23]. Vitamin E coexists with fats [21]. Therefore, we speculate that high intake of fat contributes to enhance the effect of vitamin E as an antioxidant in the inner ear to delay AHL progression in C57BL/6J mice.

**Table 1:** Ingredients in the experimental chows.

	HFD32 (100g)	CE-2 (100g)
	"High Fat Diet"	"Normal Diet"
Moisture (%)	6.2	8.8
Crude protein (%)	25.5	25.1
Crude fat (%)	32.0 *	5.1
Plos Crude fiber (%)	2.9	4.4
Crude ash (%)	4.0	6.9
Nitrogen-free extract (%)	29.4	49.7
Energy (kcal)	507.6 *	345.4
Calcium (g)	0.71	1.09
Phosphorus (g)	0.44	1.05
Magnesium (g)	0.08	0.35
Potassium (g)	0.59	0.98
Sodium (g)	0.23	0.35
Manganese (mg)	1.41	11.85
Iron (mg)	5.55	35.67
Copper (mg)	0.85 *	0.79
Zinc (mg)	6.22	6.23
Vitamin A (IU)	170	1850
Vitamin D (IU)	130	225
Vitamin E (mg)	17.8 *	6.4
Vitamin B1 (mg)	0.8	1.9
Vitamin B2 (mg)	1.0	1.2
Vitamin B6 (mg)	0.7	1.4
Vitamin B12 0028µg)	2.4	5.7
Vitamin C (mg)	-	27
Pantothenic acid (mg)	1.7	3.4
Niacin (mg)	9.6	17.8
Folic acid (mg)	0.3 *	0.2
Choline (mg)	150	200
Biotin (µg)	23.5	44.8

* More abundant components in HFD32 than in CE-2.

AHL has been shown to be delayed by certain interventions such as caloric restriction [12][24][25] and supplementation with antioxidants [23][26][27][28][29]. However, the effects of antioxidants differ depending on the strain. Antioxidants were effective in delaying AHL progression in the C57BL/6 mice [23][29]. In contrast, an antioxidant-enriched diet did not delay AHL progression in the CBA/J mice [30]. Mice of the C57BL/6J inbred strain present a genetic progressive sensorineural hearing loss and have been widely used as a model of adult-onset sensorineural hearing loss and presbycusis [11][12][13]. The C57BL/6J strain carries a specific mutation (*Cdh23* ^*753A*^) in *Cdh23*, which encodes a component of the hair-cell tip link [31][32]. The *Cdh23* ^*753A*^ mutation is known to promote the early onset of AHL in C57BL/6J mice, whereas the CBA strain does not possess the *Cdh23* ^*753A*^ mutation and displays a late onset of AHL [13][32]. Oxidative damage increases with age in the cochlea of C57BL/6J mice, and such oxidative damage plays a causal role in AHL [29]. We deduced that cochleae in mice carrying the *Cdh23* ^*753A*^ mutation are more susceptible to oxidative stress; they are also more susceptible to antioxidants. It is inferred that the different susceptibilities between C57BL/6J and CBA strain to vitamin E, as an antioxidant, caused the different results of hearing function in this study.

The HFD has caused diabetes and obesity in various strains of mice and rats. However, the effects of HFD depend on many factors, including the type, dosage, timing, and duration of HFD, and the species and strains used [33][34]. In previous reports, glucose tolerance was impaired in HFD-fed C57BL/6 mice in comparison with normal diet-fed mice [17]. However, there was no difference in either nonfasting [17] or fasting glucose levels [18] between the HFD-fed and normal-diet-fed C57BL/6 mice. These results are similar to the blood glucose levels obtained by us (Fig. 1C) and indicate that HFD feeding does not cause severe diabetes in C57BL/6 mice. On the other hand, Vasilyeva et al. reported that HFD-fed CBA/CaJ mice show apparent elevation in fasting glucose levels in comparison with normal diet-fed mice [7].

Our present results suggest that the metabolic changes caused by HFD affect the mechanisms underlying early AHL progression in C57BL/6J mice. We speculate two reasons for this positive effect of the HFD on hearing in C57BL6/J mice. The first is the antioxidant effect of vitamin E in the inner ear. Vitamin E could have prevented oxidative damage to the C57BL/6J inner ear caused by aging and HFD itself. The second is that C57BL/6J mice have less abnormal glucose tolerance by HFD than the CBA mice. It may minimize the damage from high glucose to the inner ear. Future studies will investigate the mechanism(s) for this delay in AHL progression in HFD-fed C57BL/6J mice. Any positive results in future studies could lead to new therapeutic or preventive interventions for patients with AHL.

In conclusion, HFD could delay AHL progression in the C57BL/6J mice. The elevation of ABR thresholds and loss of SGCs associated with aging were significantly suppressed in the HFD-fed group in comparison with the control group. The CBA/N-slc mice in the 12-month HFD subgroup exhibited a greater elevation of ABR thresholds and loss of SGC. Further studies are required to determine the mechanism(s) underlying the effects of HFD on AHL.
